# Trilogies: Lessons from 50 Years Facilitating Community-based Health Assessments and Planning in Appalachia

**DOI:** 10.13023/jah.0601.10

**Published:** 2024-09-01

**Authors:** Bruce Behringer

**Affiliations:** East Tennessee State University (retired) and Tennessee Department of Health (retired)

**Keywords:** Appalachia, facilitation, public health practice

## Abstract

Involvement of community and organizational groups is fundamental to most public ventures. Most social, health, economic, and educational improvements in Appalachia have been characterized by successfully integrating community input and finding ways to encourage organizational change and collaboration.

Managing group process and related facilitation skills are fundamental competencies for public health professionals and others guiding change efforts. Groups from communities and organizations can get stalled in their deliberations; a facilitator frequently must think quickly to diagnose the situation and propose alternative approaches. Creative and flexible approaches, learned through practice experiences, can blend with theories and frameworks learned in academic preparation from multiple disciplines in order to effectively encourage group progress.

Over a 50-year career (1972–2022), sets of three related concepts were formed as trilogies and used during work with groups of diverse compositions, in multiple locations, and addressing varied topics. The trilogies proved helpful in encouraging group tasks related to assessment, planning, monitoring, and evaluation. Trilogies also were deployed as a facilitation technique to pose thoughtful options as groups considered difficult issues and maneuvered through stagnant or conflict-prone situations. This paper presents twelve trilogies organized around six common group-process questions. A reference for the source of each trilogy is provided, and several Appalachian-specific examples of how trilogies were deployed are described.

## INTRODUCTION

Most people agree that community involvement enhances the quality and richness of community assessment and planning activities by inviting authenticity and complexity. Likewise, engaging community groups and gathering input from multiple organizations usually strengthens the process and outcomes of community health improvement plans. Those who work with community advisory boards, project committees, or multisector coalitions sometimes encounter situations in which the group process has slowed or stopped. Whether due to loss of focus, inertia, or conflict, help may be required to move forward. While there is no single blueprint for facilitating groups to organize their time, assess their assets and needs, or set and meet their objectives, there are some tried-and-tested techniques that may help.

Over a 50-year career (1972–2022) of working with groups of diverse compositions, mostly in Appalachia, I learned the necessity of designing plans of engagement while also being prepared to adapt those plans by offering alternative pathways for groups achieve their community development and health improvement objectives. Standing alone, in front of a stalled group, one must think quickly to diagnose the situation, adjust, and offer different approaches. This is not unlike challenges faced by medical and other health practitioners, or organizers in the education and business sectors. Theories and frameworks learned in academic preparation can blend with creativity and flexibility gained through practice experiences.^1^

An example of this blend is the use of trilogies, sets of three related ideas. These can include three categories that differentiate or better define a problem or solution, three optional perspectives from which to consider an issue, three alternative actions, or three steps in a defined process.

Several group process situations encountered over time are described below. They represent examples from work with a wide variety of group settings. These included myriad boards of directors’ meetings, professional development training with Virginia’s community health centers (CHCs), development and maintenance of community–academic partnerships between a regional Appalachian university and surrounding rural counties, and community-based participatory research activities in multiple Appalachian states. While the questions and examples emerged from work with groups, trilogies can also encourage expansive thinking, promote productive communication, and lead to improved outcomes with individuals and communities.

In the narrative below, common situations facilitators may encounter in working with groups are introduced through a leading question, and different trilogies to help further group discussions are described. Yet why trilogies? First, sets of three are usually easy to memorize and recall when facilitating group thinking. Second, posing a few questions avoids the risks inherent in asking just one or two—presenting solely one way to consider an issue may be seen as dictatorial, and two perceived as posing alternate zero-sum choices. When three options are presented, groups can usually discern differences while not becoming confused or overwhelmed. The facilitator’s challenge is not to sound too academic, while at the same time being prepared to explain how the concepts are rooted in literature or practice-based experience. The task is to identify an applicable trilogy, then introduce it to guide productive group consideration at the right time within the discussion.

### Losing focus: *Tell me again, what is the purpose of this action or program?*

During times of confusion, how can a group resurrect and reconstruct its charge to clarify its purpose? A simple tool for this is the Planning Pyramid.^2^ The Pyramid includes three key elements which can be extracted from written documents or through group discussion using a flip chart:

1. What problem(s) is (are) to be addressed?
2. What is (are) the goal(s) for change?
3. Which general strategies were selected for implementation?

The Planning Pyramid is a simple visual tool designed to define, separate, and organize the maze of ideas that arise during assessment and planning. Completed Planning Pyramids are also a good tool to orient new members or refresh an organization on a sense of its broad purpose. Ideas are portrayed in a pyramid shape. Groups usually identify multiple problems associated with an issue they are formed to address. For any singular problem, groups may identify several goals for change, which in turn could be addressed through alternative or complementary strategies. The Pyramid standardizes terminology to differentiate problem statements from goals for change. It also encourages groups to consider multiple broadly stated strategies that could impact the goal.

**Figure 1:**
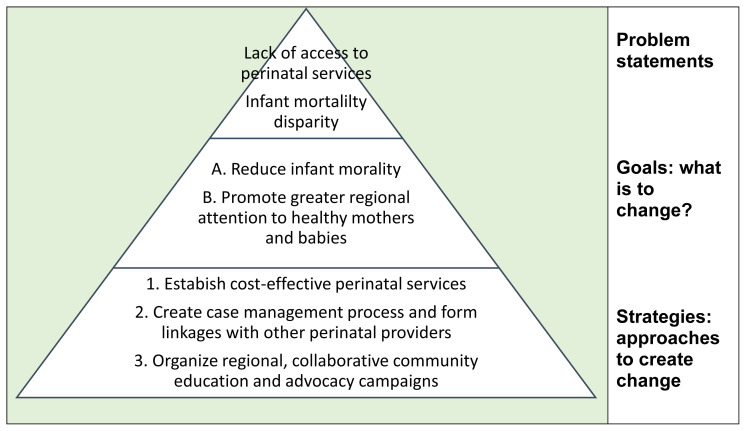
A sample Planning Pyramid for development of a women’s health center, the Central Virginia Community Health Center (1983)

In Fig. 1, input was collected about local perinatal issues from board and staff members of a rural CHC. Ideas were categorized as either Problems, Goals, or Strategies within the Pyramid. This product allowed the board of directors to weigh and sequence its priorities, then design interventions to address a mix of personal care and systemic community health issues.

A second trilogy delves deeper into the purposes of change. When group frustration reaches the “What are we really trying to do here?” level, it’s time to reassess long-term aims. A trilogy drawn from recent health education literature describes alternative intentions of health programming:^3^

1. Ensure the cultural centeredness of health programs
2. Guarantee that communities are empowered when defining and selecting problems, goals, and strategies
3. Assure expected outcomes will promote health equity and reduce health disparities

Long-term program impact is not always explicitly discussed by groups focused on short-term operations. Proposed impacts may be found in broad program statements of purpose, but then lost during cycles of annual planning, monthly monitoring, and daily implementation activities. Introducing the three ideals above can clarify and reinforce long-term intent. These concepts may also create conflict. Some stakeholders may support a program but be uneasy about open discussions of agreements that focus on culture, empowerment, or equity. One way to introduce this trilogy is through an exercise to develop program logic models which require explicit statements of long-term program impacts.^4^

### Considering alternative strategies: *What are different ways this program can work to achieve our health improvement or promotion goal?*

Groups often convene to solve problems only to find members already have favorite strategies to achieve goals. In these cases, planning discussions may lead to emotional debates over personal preferences. An organizer is challenged to create opportunities for group thinking that consider multiple, broadly stated strategies to address the same goals. Moreover, strategic thinking should not be confused with operational planning that determines specific activities. To encourage this approach, several trilogies can be used.

Rothman’s community organizing model includes a trilogy of broadly stated strategies.^5^ Groups can assess each community development approach separately, then compare the possibilities while setting program directions.

1. Locality development: pursue change through active participation of diverse people with a focus on actions that improve organizational capacities
2. Social planning: employ a technical planning and problem-solving process that engages professional experts to assist
3. Social action: organize disadvantaged peoples and communities to make demands on the larger community for increased resources or improved treatment

Implementing health improvement efforts frequently requires discussion of organizational change. Diffusion of Innovation Theory^6^ recognizes sets of factors related to adopting change, including identifying its relative advantage, amount of change required from existing procedures and policies, and costs.

**Case 1. Rothman’s Trilogy in Action**

Funding was provided by the Appalachian Regional Commission and Federal Office of Rural Health in 2005 to assist 26 communities to participate in a self-assessment and planning process addressing local substance abuse issues. A three-day invitational conference was conducted during which Rothman’s trilogy was introduced to encourage strategic thinking by community groups. Groups defined a range of different local problems and agreed on goals to reduce the prevalence of substance abuse and its impacts. Each community used Rothman’s trilogy to weigh organizing options that defined its strategy. Some chose to strengthen local coalitions (locality development), several planned to meet with provider organizations to identify possible new services (social planning), and others directly engaged elected officials and local media in discussions to improve awareness and promote community action (social action).

Another traditional business model of organizational change from Chin and Benne provides a different trilogy:^7^

1. Empirical-rational: knowledge enables change. Provide new information about how a proposed change is beneficial to the organization and empowers leaders to pursue change as they become aware of their self-interest.
2. Normative-re-educative: change organizational norms. Leaders set new orientations, standards, or guidelines that commit to strategies like valuing partnerships, community involvement, and setting and measuring improvement goals.
3. Power-coercive – require compliance of those with less power to policies enacted by those with greater power. Organizations frequently use incentives and disincentives to encourage internal change that supports meeting their goals.

### Thinking through problems and solutions: *Is it time to break out traditional health education and behavior theories?*

Groups can be very impressed, and sometimes relieved, to learn that others have already thought about their issue. Whether the aim is to organize a list from seemingly unrelated factors or to generate additional ideas, traditional health education and behavior theories and models can be very useful. They appear in easy-to-apply trilogies found in traditional texts and internet sites.^8^,^9^ Three easy-to-remember trilogies are starters that can open the door to lively group discussions about the theory of a problem.

Reviewing three factors that influence behavior change is a good starting point.^10^ This trilogy draws on cognitive-behavioral theory used by health and behavioral health care practitioners. Breaking down discussions about behavioral goals using this trilogy introduces a thought process that illustrates the complexity of behavior change.

1. Awareness and knowledge: behavior is mediated by cognition. Awareness is a first step for people to gain knowledge that may affect how they act.
2. Beliefs/attitudes: knowledge is necessary for, but not sufficient to produce, most behavior changes. Motivation to perform a behavior is influenced by beliefs and attitudes.
3. Behaviors/habits: behavior change may result from new knowledge and beliefs. However personal, social, and environmental factors are key to adopting skills and promoting new habits.

The PRECEDE–PROCEED model^11^ offers another trilogy to analyze and promote individual, group, or community behavior change. These simple factors can be introduced to guide discussions and are particularly beneficial when working with groups not aware of behavior change theories or models. Many iterations of worksheets and exercises are available to assist in capturing the factors:^12^

1. Predisposing factors: presence of knowledge, attitudes and beliefs, along with intentions and demographics
2. Enabling factors: personal skills, resources, social and physical environment that influence behaviors
3. Reinforcing factors: program actions, such as reminders, reinforcements, and social support

While slightly more complex, the Health Belief Model^8^ is another tested tool for understanding and promoting preventive health behaviors. The Model first explored a clinical issue: how to encourage greater acceptance of vaccinations. There are more than three constructs in the Model, but the set of three ideas below has been demonstrated to promote intense discussion about personal health behaviors and adoption of preventive actions. A facilitator can lead the group to consider each idea, weigh its potential as a focus, and then design intervention strategies for change.

1. Perceived susceptibility: an individual’s perceived vulnerability to the threat of disease
2. Perceived severity: belief in the negative consequence of the related disease
3. Benefits v. barriers: weighing positive benefits of action with perceived barriers to action

**Case 2. The Health Belief Model as a trilogy**

The Centers for Disease Control and Prevention invested grant funds through a consortium of two universities and a regional health district to document and describe Appalachian regional cancer disparities beginning in 2001. One project engaged groups in nine diverse communities in rural East Tennessee, Southwest Virginia, and Eastern Kentucky. Community focus groups were organized, and each group identified what was commonly known, unknown, and uncertain about one of four types of cancer. Concepts from the Health Belief Model were a key element used to frame focus group discussions. Facilitators helped community groups describe their own senses of susceptibility to and severity of cancers, and then identify common community concerns about the benefits and barriers to seeking preventive screenings and treatments.

### Focusing on evaluation: *What results should we expect and how can we measure effects of our program?*

Groups charged with health and community improvement efforts often gravitate into two camps. One focuses intently on activities planning, budgeting, and counting units of service. Others take a broader view, concentrating on how the theory and design of programs are expected to address problems. Encouraging either of these groups to think more comprehensively can be done by deploying two trilogies.

Logic Models^4^ can be a planner’s and evaluator’s best friend. So much attention can be focused on resources and activities for the planned work that consideration of intended results might be slighted. This tendency can be countered through developing a standard program logic model, which requires the sequential display of operational elements and outcomes. The final three steps of the logic model are outputs (countable results of activities), outcomes (desired effects achieved from intervention activities), and impacts (expected long-term results of change). Many groups found this trilogy useful at their beginning planning steps, then repeated the exercise over time to recognize changes and achievements.

Complementary to logic models is a discussion of categories and timeframes for collecting evaluation data. For groups new to evaluation design, another trilogy differentiates traditional types of evaluation and emphasizes the importance of continuous attention to data collection.^13^

1. Formative evaluation: gather information during program operations to use for continuous improvement and detection of ways to strengthen program implementation
2. Process evaluation: track and understand how the program actually operates through studying program policies and processes, as well as tracking effects of key events and trends
3. Summative evaluation: review data at program completion to compare achievements with intended outcomes. Summative evaluations can effectively integrate important interpersonal, individual, organizational, and community influences and outcomes. Results of these discussions can enhance understanding of the program’s worth.^14^

### Engaging the right people in the process: *What types of people would be beneficial to recruit?*

Perhaps the most important element of working with groups is a thoughtful approach to engaging the right people. Jim Collins’ book *Good to Great* calls this getting the right people on the bus.^15^ Three different trilogies can help groups characterize existing membership and consider recruitment of new members who might improve group effectiveness.

The following categories of individuals who volunteer were drawn from reflections on many years of board of directors training with CHCs and public health advisory groups.^2^ Each broad characterization is based on personal motivations that drive involvement and commitment of people to improvement programs. Program success relies on involving the right mix of people from all three categories of interests:

1. Power brokers: people who serve to have a voice in group actions. These persons frequently represent existing governing or affiliated organizations.
2. Socialites: people with deep social networks. Participation in groups often represents just one of many personal connections or professional memberships in their community.
3. Users: people who will directly or indirectly benefit or are harmed if services are sustained or eliminated. They demonstrate a strong personal stake in program success.

Another applicable trilogy can be found in Malcolm Gladwell’s book, *The Tipping Point*.^16^ He differentiates people with special skills needed to “tip” adoption of an innovative idea or program into action:

1. Connectors: people who know people. Like socialites, they have broad networks and honed communication skills that enable them to reach the right influential people who can move ideas along.
2. Mavens: people who accumulate knowledge. These are formal and informal information specialists able to connect with new information, effectively package it, and pass it along.
3. Salespersons: people with skills to persuade. They can combine the appropriate rationales with persuasive arguments for different audiences to encourage change.

The final trilogy comes from *Switch: How to Change Things When Change Is Hard*.^17^ The authors employ the analogy of the difficult task of riders trying to get elephants to follow a desired path. Human psychology principles are used to describe, then compare, each element in their story with characteristics of people who can bring about change with individuals, companies, and communities:

1. Direct the Rider: involve the rational side of people. These are clear thinkers who can point to what success means, communicate the destination, and direct critical moves.
2. Motivate the Elephant: engage the emotional side. Some people seem to naturally be able to encourage change without force. They emphasize the importance of personal involvement, value of cooperation, and interpret any needed change as small but significant.
3. Shape the Path: understand how the situation and broader environment influence potential program success. These are people who see the big picture of the proposed program and promote new paths of habits and behaviors that act to “rally the herd” in the direction of change.

### Defining your role: *And what is it that I am doing here?*

Facilitators find themselves playing different roles with different groups at different times. They can be group members; selected, elected, or appointed leaders; or even consultants. A trilogy from Schein’s book *Process Consultation*^18^ explains three clearly different roles one can be play. This set of ideas was used to confirm expectations and establish boundaries on types of assistance whenever I would begin to work with groups:

1. Purchase model: provide specific content expertise or a professional service designed to accomplish a task. Examples included organizing and conducting strategic planning workshops, managing community meetings, and producing reports to address a defined community and organizational issue.
2. Doctor–patient model: engage in a diagnosis to identify problems and recommend actions. Topics and recommendations vary depending on the facilitator’s content expertise. For health issues, this included introducing and deploying different interactive community assessment methods, then interpreting results with the group and sponsoring organization.
3. Process consultation: support the group to perceive and improve its own work processes. I frequently attended and analyzed board meetings to assess how ideas and issues were generated and resolved, identified ways to improve group communication, demonstrated how to set agendas, and suggested methods to align board action with organizational plans.

**Case 3. Using Schein’s Trilogy to Structure Community Partnerships**

A Community Partnership Program provided an opportunity for an Appalachian university to collaborate with regional Hispanic and African-American coalitions.^1^ Partnerships developed community-based learning projects for students that addressed coalition-identified issues. For the Hispanic group, the first priority was to identify opportunities, then encourage community members to access college enrollment incentive programs. For the regional coalition led by Black leaders, Diabetes prevention and management was the top priority. Schein’s trilogy provided a framework for each coalition to define what they wanted the university to give as part of the partnership. Hispanic leaders requested process consultation assistance. The facilitator helped the coalition organize agendas and recruited regional public schools, elected and business leaders, and university administrators to attend meetings sponsored and managed by the coalition. Black leaders requested “doctor–patient” consultation for health science faculty to help document diabetes-related disparities of minority populations and then collaborate to plan multiple community-based prevention and treatment interventions.

## SUMMARY

Accomplishing change with and through groups is both fun and challenging. An ability to draw upon one’s academic background and professional experience should be tempered with an intellectual humility that prioritizes preparation, listening, and seeking group acceptance and direction.^19^ Using trilogies can be a helpful group process technique for leaders and facilitators, or for others charged with assisting individually focused change. There is a risk that, while employing theoretical concepts may impress upon some group members an organizer’s professional acumen, overreliance on trilogies may paint the facilitator as “an academic.” Finding the balance comes through experiences of diagnosing groups, their purposes, and how best to offer assistance that will be most valuable. Storing a repertoire of trilogies for use at the right time, with the right issue, and the right group can prove an exciting testament to a practitioner’s skill and ability.

Over time I have no record keeping track of the use of the wide variety of trilogies described above. The trilogies were deployed, either as part of planning or as an adaptive response to situations in highly diverse settings, with many types of groups, and on an endless array of health, education, and social issues. Former students, colleagues, and organizations continue to use trilogies they observed as successful in action. Were all the attempts to guide communication and clarify thinking successful? No, but there is a positive career record of getting and keeping people engaged and progressing toward their goals, due in part to the flexible and responsive trilogies noted here.

## Footnotes

1 The Coalition identified itself as “African-American.” The leaders self-identified as Black. These self-identifications have been retained here.

## References

[1] Henderson HL Sendall MC Positioning the scholarship of teaching and learning firmly in the center of health promotion pedagogy Pedagogy Health Promot 202 8 1 6 8 10.1177/23733799211061281

[2] Behringer B Involving your board of directors in your health center: Tricks from the Front Line Virginia Primary Care Association 1991 Copy available at: http://lib.ncfh.org/pdfs/2016/2595.pdf Accessed Apr. 2, 2024

[3] Kegler MC Wolff T Christens BD Butterfoss FD Francisco VT Orleans T Strengthening Our collaborative approaches for advancing equity and justice Health Educ Behav 2019 6 1_suppl 5S 8S 10.1177/1090198119871887 31549552

[4] W. K. Kellogg Foundation Logic Model development guide Jan 2004 Available at: https://www.wkkf.org/resource-directory/resources/2004/01/logic-model-development-guide

[5] Rothman J Planning and organizing for social change: Action principles from social science research New York Columbia University Press 1974 13:978-0-2310-3774-7

[6] Rogers EM Diffusion of innovations 5th Edition New York Simon and Schuster 2003 13:978-0-7432-5823-4

[7] Chin R Benne KD General strategies for effecting changes in human systems Bennis WG Benne KD Chin R The planning of change Austin TX Holt, Rinehart & Winston 1985 13:978-0-0306-3682-0

[8] Rimer B Glanz K Theory at a glance: A guide for health promotion practice 2nd edition Bethesda MD National Cancer Institute 2005 13:978-0-3592-4434-8

[9] Celeste N What is behavior change in psychology? 5 models and theories PositivePsychology.com Aug 14 2021 Available at: https://positivepsychology.com/behavior-change/#:~:text=1%20Reducing%20procrastination%202%20Incorporating%20regular%20selfcare%20activities,4%20Going%20to%20bed%20earlier%205%20Practicing%20mindfulness Accessed Apr. 2, 2024

[10] Fisher JD Fisher WA Changing AIDS-risk behavior Psychol Bull 1992 111 3 455 74 1594721 10.1037/0033-2909.111.3.455

[11] Green LW Kreuter MW Health program planning: An educational and ecological approach New York McGraw-Hill Education 2005 13:978-0-0725-5683-4

[12] Orlowski MA Hallam J PER Worksheet Available at: https://medicine.wright.edu/sites/medicine.wright.edu/files/page/attachments/PER.pdf

[13] Centers for Disease Control and Prevention Division of SDT Prevention Types of evaluation Available at: https://www.cdc.gov/std/Program/pupestd/Types%20of%20Evaluation.pdf

[14] McLeroy KR Bileau D Steckler A Glantz K An ecological perspective on health promotion programs Health Educ Q 1988 15 351 77 10.1177/109019818801500401 3068205

[15] Collins J Good to great New York Harper-Collins Publishers 2001 13:978-0-0666-2099-2

[16] Gladwell M The tipping point Boston Little Brown and Company 2000 13:978-0-3163-1696-5

[17] Heath C Heath D Switch: How to change things when change is hard New York Broadway Books 2010 13-978-0-3855-2875-7

[18] Schein EH Process consultation revisited Reading, MA Addison-Wesley 1999 13-978-0-2013-4596-4

[19] Johnson SS Editor’s desk: It’s critical to cultivate intellectual humility Am J Health Promot 2022 36 8 1399 1401 10.1177/08901171221125326a 36305506

